# Determination of the embedded electronic states at nanoscale interface via surface-sensitive photoemission spectroscopy

**DOI:** 10.1038/s41377-021-00592-9

**Published:** 2021-07-27

**Authors:** Hui-Qiong Wang, Jiayi Xu, Xiaoyuan Lin, Yaping Li, Junyong Kang, Jin-Cheng Zheng

**Affiliations:** 1 Fujian Provincial Key Laboratory of Semiconductors and Applications, Collaborative Innovation Center for Optoelectronic Semiconductors and Efficient Devices, Department of Physics, Xiamen University, Xiamen, 361005 China; 2 Department of Physics, Xiamen University Malaysia, Sepang, 43900 Malaysia; 3 Department of New Energy Science and Engineering, Xiamen University Malaysia, Sepang, 43900 Malaysia; 4 College of Science, Henan University of Technology, Zhengzhou, 450001 China

**Keywords:** X-rays, Electronics, photonics and device physics

## Abstract

The fabrication of small-scale electronics usually involves the integration of different functional materials. The electronic states at the nanoscale interface plays an important role in the device performance and the exotic interface physics. Photoemission spectroscopy is a powerful technique to probe electronic structures of valence band. However, this is a surface-sensitive technique that is usually considered not suitable for the probing of buried interface states, due to the limitation of electron-mean-free path. This article reviews several approaches that have been used to extend the surface-sensitive techniques to investigate the buried interface states, which include hard X-ray photoemission spectroscopy, resonant soft X-ray angle-resolved photoemission spectroscopy and thickness-dependent photoemission spectroscopy. Especially, a quantitative modeling method is introduced to extract the buried interface states based on the film thickness-dependent photoemission spectra obtained from an integrated experimental system equipped with in-situ growth and photoemission techniques. This quantitative modeling method shall be helpful to further understand the interfacial electronic states between functional materials and determine the interface layers.

## Introduction

### Important roles of interfaces

An interface in physical science or materials science is usually defined as the boundary between two different materials or different physical states of the same matter. Interfaces can be classified into several typical catalogs, such as solid–gas interface, solid–liquid interface, liquid–liquid interface, and solid–solid interface, and so on. Surface is a special interface, formed between material and vacuum. The interface is important and interesting because its properties and behavior can be quite different from the adjacent bulk phases. The emergence of the interesting phenomena is resulted from the thermodynamic constraints enforced by the two-dimensional surface/interface. The different concentrations (or density) and structural arrangements of atoms or molecules in the surface/interface region compared with bulk materials, result in unique physical and chemical properties of interfaces. Interfaces, therefore, are often found to play a central role both in nature and within a variety of different technological applications, devices, and industrial processes^1^. For example, *solid–gas interfaces* have been involved in catalytic reactions such as the reduction of harmful gas emissions in catalytic converter in automobiles, producing industrial chemicals through heterogeneous catalytic reactions, thin-film growth during microelectronics processing, etc. *Liquid–gas interfaces* are important for environmental problems. *Liquid–liquid interfaces* play a large role in the biological process and many daily life applications including detergents, foods, and paints by the stabilization of emulsions and micro-emulsions. *Solid–solid interfaces* are especially important in advanced functional materials and devices^1,2^.

The nanoscale interfacial properties between functional materials can significantly affect a wide range of device characteristics, especially for modern microelectronics. Such effect would either hinder the performance of electronics or actually open opportunities for innovative design of new type of devices. For example, transition metal oxides, which exhibit rich material properties due to the unique characteristics of their outer *d* electrons, are promising for the next-generation oxide electronics^2–5^. Both atom reconstruction and electron reconstruction, as well as spin, orbital, and charge coupling at the oxide interfaces have led to novel interface physics as well as emergent phenomena^6–10^. The conductivity, as well as superconductivity observed at the interface between the two wide-bandgap insulators of LaAlO_3_ and SrTiO_3_, are remarkable examples^8,11–15^. Heterojunctions that hybrid with semiconductors have also demonstrated significant roles in photocatalysts^16–18^. It is thus of great importance to characterize the electronic states at the interface.

### Characterization of interfaces

Because of the importance of interfaces, many different tools or methods have been developed to characterize the chemical composition, geometrical arrangements, and various properties, including mechanical properties and processes (such as thickness, roughness, clusters/particles dimensions, and distribution, friction, fracture, strength, strain, stress, deformation properties, fatigue resistance, wear, etc.), physical properties and processes (e.g., density, crystallization, physical inter-diffusion, dielectric and magnetic properties, energy density, etc.), chemical properties and chemical processes (e.g., elemental and molecular compositions of the layers, size, and orientation of individual molecules, adhesion, corrosion, passivation, interfacial interactions, chemical diffusion, barrier properties, etc.), as well as optical properties and processes (including refractive indices, spectral reflectivity, and transmittance, optical absorption properties, etc.)^19^. The characterization methods can be classified into different groups regarding the properties of objects studied, or detection features of tools. The classifications of the surface and interface analyzing techniques are available in several reviews and books^19–23^. Following the classification presented in refs. ^19,20^, we listed the main surface and interface analysis techniques in Table 1, according to the detection features. Typical analytical methods for buried interfaces include electron detection methods, photon detection methods, neutron detection methods, ion detection methods, and scanning probe methods.

**Table 1:** List of surface and interface analyzing techniques^19,20^ according to their detection features

Photon detection methods	Electron detection methods	Neutron detection methods	Ion detection methods
TXRF and standing wave XRF, Energy dispersive and wavelength-dispersive XRF, Glancing-incidence (GI)-X-ray reflectivity (XRR), GI-X-ray diffuse scattering, GI-resonant X-ray scattering, GI-XRD GI-XAFS, OES, Laser ablation or sputter depth profiling, Ion-beam spectrochemical analysis, RAIRS, ATR, SEIRA, ATR-FTIR, Surface Raman spectroscopy, Optical reflectivity and ellipsometry, SFG, SXAPS, IPES	XPS, AES, EELS, APS, SEM, EF-TEM, STM, LEED	Neutron reflectivity Neutron diffraction and scattering	SIMS, Electron impact (EI)-SNMS, Laser-SNMS, RBS, LEIS, ERDA, NRA, FIM

Please refer to Appendix 1 for the explanation of acronyms

### Typical analytical methods for buried interfaces

**Fig. 1:**
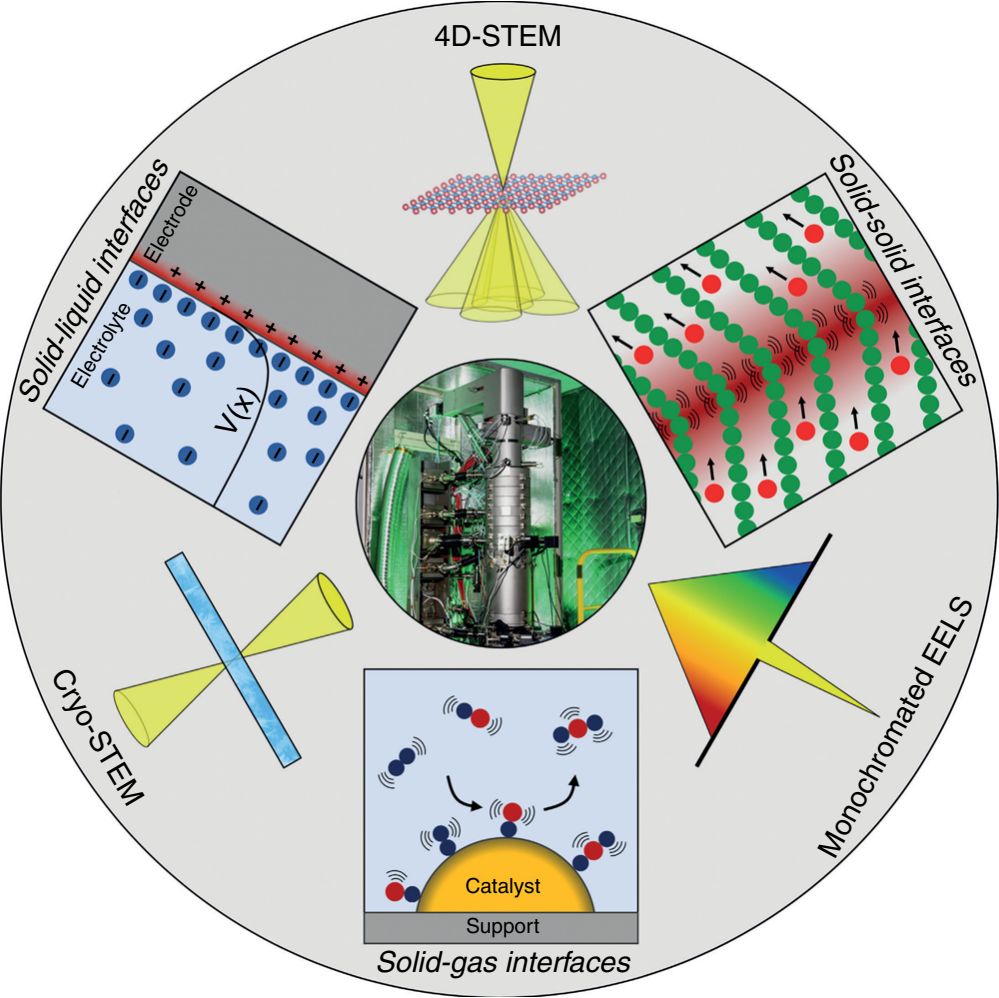
Overview of probing functional interfaces in energy materials using emerging electron microscopy techniques. Reproduced with permission^24^. Copyright 2019, Wiley

Emerging electron microscopy techniques, based on scanning transmission electron microscopy (STEM) and/or electron energy loss spectroscopy (EELS) (such as 4D-STEM, cryo-STEM, and monochromated EELS) are very useful tools for probing functional interfaces in energy materials (as shown typically in Fig. 1)^24,25^. Spatially resolved EELS is capable of examining the conduction band structure and has been used to study the electronic changes at perovskite oxide heterointerfaces^7,26,27^. However, EELS is usually equipped with the expensive facility of STEM and is also limited by the time-consuming, destructive sample preparation necessary for generating electron transparent specimens. Cathodoluminescence (CL) is capable of probing the emission properties at the interface area^28,29^. Photoemission spectroscopy (PES), including X-ray photoemission spectroscopy (XPS) and Ultraviolet photoemission spectroscopy (UPS) are powerful to investigate the valence band structure while X-ray absorption spectra (XAS) is frequently used for conduction band investigation. However, they are usually classified as “surface sensitive techniques”, due to the limitation of electron mean free path. This review aims to introduce the development and extension of these techniques to probe the buried electronic states at the interface.

## Determination of interface electronic states using photoemission spectroscopy

In general, there are three approaches to extend the application of “surface sensitive” photoemission spectroscopy to study buried interfaces: (1) Tuning the photon energy; (2) Adjusting the probing angle with respect to the surface normal; (3) Capturing thickness-dependent photoemission spectra. In this section, we will review the use of hard X-ray photoemission and resonant soft X-ray angle-resolved photoemission for probing interface electronic states, which increase the profiling depth by increasing the photon energy. A quantitative modeling method will then be introduced to extract the buried interface electronic states based on thickness-dependent photoemission spectra.

### Hard X-ray photoemission spectroscopy

**Fig. 2:**
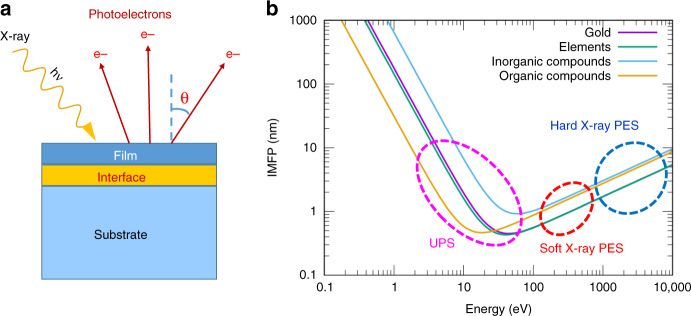
Probing interface by adjusting the probing angle or tuning the photo energy. **a** Depth profiling by angle-resolved X-ray photoemission spectroscopy. **b** The inelastic mean free path (IMFP, *λ*) of photoelectron with different kinetic energy ranges indicated. (The IMFP plots are generated using formulas 5 and parameters 1 reported by Seah and Dench^32^, namely, $\lambda = \frac{{177}}{{E^2}} + 0.054\sqrt E$ for “Gold”, $\lambda = \frac{{143}}{{E^2}} + 0.054\sqrt E$ for “Elements”, $\lambda = \frac{{641}}{{E^2}} + 0.096\sqrt E$ for “Inorganic Compounds”, and $\lambda = \frac{{31}}{{E^2}} + 0.087\sqrt E$ for “Organic Compounds”. The units of *λ* and *E* are nm and eV, respectively.)

While the photon energies of X-rays used in the regular research laboratories for photoemission spectroscopy are usually limited to 1486.7 eV (Al Kα radiation) or 1253.6 eV (Mg Kα radiation), the development of synchrotron radiations has made it possible to tune the photon energies in a wide spectral range from infrared to hard X-rays^30^. The maximal probing depth is defined as 3*λ*cos*θ*, where *λ* is the effective inelastic mean free path (IMFP), and *θ* is the angle between the detection direction and the surface normal (as shown in Fig. 2a)^31^. The mean free path of photoelectrons escaping from the solid as a function of kinetic energy^32,33^ is shown in Fig. 2b.

The angle-dependent XPS as a nondestructive method was often used for the characterization of chemical composition and electronic structure of ultrathin layers such as tin oxide films^34^, heterostructures between functional oxides^35^ or semiconductors^36–38^. The angle-dependent hard x-ray photoemission spectroscopy (with *hv* = 3 keV) has been performed at Berlin Electron Storage Ring Society for Synchrotron Radiation to analyze the depth-profiled interface electron gas of LaAlO_3_/SrTiO_3_ heterostructures, and the results supported an electronic reconstruction in the LaAlO_3_ overlayer as the driving force for the 2D electron gas (2DEG) formation^35^. Using hard XPS, the impact of oxygen on the band structure at the Ni/GaN interface was revealed^36^, the band edge profiles at the semiconductor heterostructures were extracted^37^, and the core-level shifts at the buried GaP/Si(001) interfaces were reported^38^. Aforementioned typical studied cases already demonstrated the powerful capability of hard XPS for buried interface analysis. A recent study further showed that the detection of deeply buried layers beyond the elastic limit can be enabled by inelastic background analysis, which demonstrated the potential for the characterization of deeply buried layers using synchrotron and laboratory-based hard XPS^39^.

### Resonant soft X-ray angle-resolved photoemission spectroscopy

Although hard XPS is capable to provide valuable and even quantitative information on the electronic properties at interface, it still has some drawbacks. For example, the evidence of 2DEG at oxide heterointerface is indirect since the states at the Fermi level actually hosting the mobile electrons cannot be observed due to the small cross-sections of the photoabsorption at high photon energies, and thus the photoemission signals at the Fermi level are usually unfortunately missing. However, by exciting with photons tuned to an appropriate absorption edge, the resonant photoemission allows for a selective enhancement of the emission from orbitals with a given symmetry. Therefore, a combination of soft X-ray angle-resolved photoemission spectroscopy (ARPES) with resonant photoexcitation can overcome this limitation, which has been demonstrated recently^40–44^.

The electronic structures of materials are characterized by three parameters of the electrons therein, namely, energy (*E*), momentum (*k*) and spin (*s*). ARPES is often used to investigate the k-space electronic structures of buried interfaces. In a resonant ARPES experiment, polarization-controlled synchrotron radiation was used to map the electronic structure of buried conducting interfaces of LaAlO_3_/SrTiO_3_^45^. By combining X-ray photoelectron diffraction and ARPES, the interplay between electronic and structural properties in the Pb/Ag(100) interface has been studied^46^. A critical thickness for the 2DEG formation in SrTiO_3_ embedded in GdTiO_3_ was observed by resonant ARPES^47^. Electronic structure of a buried quantum dot system (In, Mn)As, grown by molecular beam epitaxy, was investigated by soft-X-ray ARPES, which combines its enhanced probing depth with elemental and chemical state specificity achieved with resonant photoexcitation^48^. Using the similar soft-X-ray ARPES technique, the electronic structure of the buried EuO/Si interface with momentum resolution and chemical specificity was probed^49^. The electronic structure measurements of the buried LaNiO_3_ layers in (111)-oriented LaNiO_3_/LaMnO_3_ superlattices^50^, the buried SiO_2_/SiC interface^51^, and the investigation of electronic phase separation at the LaAlO_3_/SrTiO_3_ interfaces^52^, were reported using soft X-ray ARPES. By combining ARPES with soft X-ray standing-wave excitation, Gray et al.^53^ provided a detailed study on the buried interface in a La_0.7_Sr_0.3_MnO_3_/SrTiO_3_ magnetic tunnel junction. A recent topical review on ARPES studies of low-dimensional metallic states confined at insulating oxide surface and interfaces was reported by Plumb and Radovic^54^.

### Thickness dependent photoemission spectra

It is also a common approach to capture a series of photoemission and/or absorption spectra as a function of film thickness and track the evolution of the spectrum features. Diebold et al.^55^ detected a crystalline ternary MnTiO_x_ at the interface of Mn/TiO_2_ by the PES and XAS. The Mn/TiO_2_(110) was observed to consist of the reduced Ti cations and oxidized Mn overlayer atoms when deposited at 25 °C, while the interfacial Ti cations in the Mn films which were annealed to ∼650 °C were found to be re-oxidized to the Ti^4+^ state and the interfacial local order was enhanced at the same time. Gao et al. identified a Fe/Si interfacial layer which was due to the chemisorption of the first Fe layer on the Si substrate, through the study of the thickness dependence of the Fe absorption signal on the substrate from in situ XAS measurements^56^. The electronic structure of the TiO_2_–Al_2_O_3_ interface was also investigated by the detailed analysis of the XAS Ti 2p spectra as a function of the TiO_2_ deposition on Al_2_O_3_^57^, revealing the formation of Ti-O-Al cross-linking bonds at the interface, which could be attributed to the significant lowering of the crystal field of Ti atoms at the interface. A characterization technique based on the atomic core-level shifts was proposed by Holmstrom et al.^58^ to analyze the interfacial quality of the layered structures; high kinetic-energy photoelectron spectroscopy with longer mean-free paths was also used to capture signals from the embedded interface layers. Gonzalez-Elipe and Yubero^59^ investigated chemical states and bonding configurations at the interfaces, mostly by probing the Auger parameter measured by X-ray photoemission.

However, there was usually a lack of presentation of the spectra of interface states that were distinguished from and excluded the contributions from the overlayer and the substrate. In the following section, we will review a quantitative modeling method to retreat the interface state spectra and determine the interface layer structure, using UPS spectra. UPS is one of the probes that are sensitive to the electronic density-of-states near Fermi level^60^, which are responsible to transfer charge along and across interfaces in device applications.

## Quantitative modeling of photoemission spectra for interface states

### Requirement of experimental setup

**Fig. 3:**
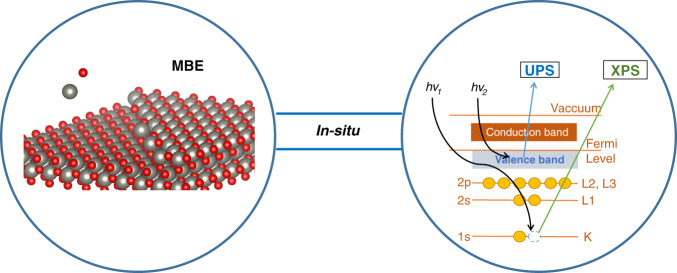
Integrated ultra-high vacuum system for the quantitative modeling of photoemission spectra for interface states. Left: Thin film growth techniques (e.g., MBE). Right: In-situ photoemission spectroscopy (XPS and/or UPS)

The quantitative probing method is to compare experimental PES spectra to model spectra as one material is grown on another. The experimental setup thus requires an integrated ultra-high vacuum (UHV) system that is equipped with the integration of thin-film growth techniques and spectra characterization probes, as shown in Fig. 3. A variety of thin film deposition techniques have been developed to meet the requirement of the high demand of different thin-film materials, including molecular beam epitaxy (MBE)^61–66^, pulsed laser deposition^67–70^, metal-organic chemical vapor deposition^71–73^, atomic layer deposition^74,75^, etc. Oxide MBE has been particularly developed to fabricate novel oxide heterostructure and superlattice^76^. A typical oxide MBE chamber is usually equipped with different evaporation metal sources from either effusion cell or e-beam evaporator as well radio frequency plasma source to generate active oxygen atoms. Quartz crystal microbalance (QCM) would be adopted to determine the flux rate of the metal evaporation sources. In situ and real-time reflection high energy electron diffraction (RHEED)^77–79^ is chosen to precisely monitor the atomic layer growth as the pattern intensity oscillates simultaneously with the surface morphology during the growth. By comparing the RHEED patterns appearing at various zone axes, the symmetry of the surface structure can be determined; and by comparing those from the substrate and from the film, the registry relationship can be speculated. A differential pumping for the RHEED gun (electron source) is needed to prevent cathode filament degradation in the high partial oxygen pressure during oxide growth. During growth, the chamber is usually cooled by running water or liquid nitrogen.

By integrating the thin film growth system with spectroscopy characterization techniques connected under UHV channels, the fleshly grown thin films then have the privilege to undergo the characterization of electronic properties without exposing to the air, thus keeping the natural features produced during growth. For the in situ characterization of XAS spectra that requires the tuning of photon energy, it would be necessary to attach a thin film growth chamber on the beam station of synchrotron radiation light source facility^80,81^. In regular laboratories, one can combine the thin film growth chamber with the analysis chamber that contains PES probing techniques^82^. For the PES experiment using the XPS instrument, X-rays with photon energy more than 1 kV are generated by bombarding either magnesium or aluminum anodes with high-energy electrons. For the PES experiment using UPS equipment, ultraviolet photons are produced using a gas discharge lamp. Helium gas is usually used to emit photons with energies of 21.2 eV (He I) and 40.8 (He II). Recently, oxide MBE growth systems have been combined with angle-resolved photoemission to prompt the new research stage of strongly correlated materials^83–90^.

### Quantitative modeling of experimental spectra

Even though PES spectra are considered surface sensitive, the mean-free path, *λ*, of the photoelectrons is large enough that the spectra will sample several monolayers into the sample. For thin films, the measured spectra will then consist of a superposition of emission from the substrate, from any interfacial states that may be present, and from the film, with each weighted by electron escape depths. The detailed analysis procedures are listed as follows.

#### Step 1: (Assuming no interface states)

**Fig. 4:**
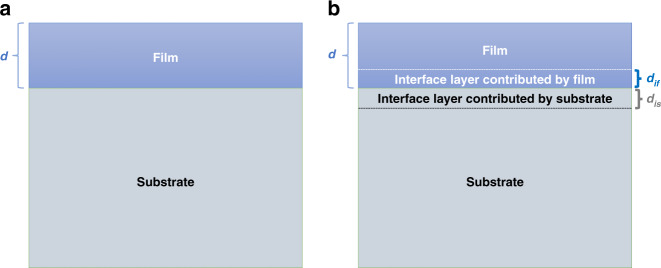
The sketch models used for the quantitative simulation of spectra. **a** A film with a thickness of *d* grown on the substrate, assuming no interface states. **b.** A film with a thickness of *d* grown on the substrate, considering interface states. *d*_*if*_ and *d*_*is*_ are the thickness if the film and the substrate, respectively, involved to form the interface layer

Before examining any possible interface states, we first assume that there are no interface states present. We then compare the measured spectra to the model spectra consisting of a superposition of spectra from the substrate and that from the film. Assuming layer-by-layer growth, and taking into account the electron escape probability $e^{ - \frac{d}{\lambda }}$ ^91^, the spectral intensity *I* as a function of thin-film thickness *d* (as shown in Fig. 4a) can be calculated as^92–94^

1 $$I_{without{\kern 1pt} interface}^{model}\left( d \right) = I_0^{Substrate}e^{ - \frac{d}{\lambda }} + I_0^{Film}\left( {1 - e^{ - \frac{d}{\lambda }}} \right)$$

$I_0^{Substrate}$ and $I_0^{Film}$ represent the experimental intensity of the “bulk spectra” of the substrate and the film, respectively. In the previous reports^92–94^, the spectral intensity of the thickest film (usually not thinner than 20 monolayers) was used as the “bulk” spectrum. Here, we further take into account the thickness *D* of the thickest film and $I_D^{Film}/\left( {1 - e^{ - \frac{D}{\lambda }}} \right)$ ^56^ is adopted to represent the intensity of the “bulk” spectrum instead. Thus Eq. (1) is modified as

2 $$I_{without{\kern 1pt} interface}^{model}\left( d \right) = I_0^{Substrate}e^{ - \frac{d}{\lambda }} + I_D^{Film}\left( {1 - e^{ - \frac{d}{\lambda }}} \right)/\left( {1 - e^{ - \frac{D}{\lambda }}} \right)$$

The IMFP *λ* in Eq. (2) can be estimated using the plots in Fig. 2b or the formulas therein. It can also be calculated based on the reports by Tanuma et al.^95,96^. The thickness *d* of the thinner film can be determined on the attenuation of the core-level photoemission line from the substrate^97^:

3 $$d = - {\it{ln}}\frac{{I_{after}}}{{I_{before}}}$$

where $I_{before}$ and $I_{after}$ are the spectral intensities of the XPS core-level photoemission line from the substrate before and after the thin film deposition with a thickness *d*. The thickness *D* of the thickest film can be probed using a variety of techniques, including microscope.

Difference spectra are then taken between the experimental $I^{expt}$ and model spectra $I_{without{\kern 1pt} interface}^{model}$:

4 $$\varDelta I\left( d \right) = I^{expt}(d) - I_{without{\kern 1pt} interface}^{model}(d)$$

If there are no obvious features in the difference spectra $\varDelta I\left( d \right)$, an electronically sharp interface without additional electronic state could be claimed.

#### Step 2: (Considering interface states)

Any difference features between the measured and model spectra may then result from the interfacial electronic structure. If an interface state exists, Eq. (2) can be changed to be: [modified from refs. ^92,93^

5 $$\begin{array}{ll}I_{with{\kern 1pt} interface}^{model}(d) = I_0^{Substrate}e^{\frac{{ - \left( {d + d_{is}} \right)}}{\lambda }} + I_D^{Film}\frac{{1 - e^{\frac{{ - \left( {d - d_{if}} \right)}}{\lambda }}}}{{1 - e^{\frac{{ - D}}{\lambda }}}}\\ \qquad\qquad\qquad\qquad+ \,I_0^{Interface}\left[ {1 - e^{\frac{{ - \left( {d_{is} + d_{if}} \right)}}{\lambda }}} \right]e^{\frac{{ - \left( {d - d_{if}} \right)}}{\lambda }}\end{array}$$

where *d*_*is*_ and *d*_*if*_ are the thickness of the substrate and the deposited film, respectively, involved to form the interface layer (as shown in Fig. 4b); *I*_*0*_^*Interface*^ is the spectral intensity for the interface layer, assuming a semi-infinite slab having the interface electronic structure; and *d* is the deposited thickness of the film. With the assumption that the experimental spectra contain interface states, we can use $I^{expt}$ for $I_{with{\kern 1pt} interface}^{model}$ in Eq. (5). Therefore, Eq. (5) becomes

6 $$\begin{array}{ll}I^{expt}(d) = I_0^{Substrate}e^{\frac{{ - \left( {d + d_{is}} \right)}}{\lambda }} + I_D^{Film}\frac{{1 - e^{\frac{{ - \left( {d - d_{if}} \right)}}{\lambda }}}}{{1 - e^{\frac{{ - D}}{\lambda }}}}\\ \qquad\qquad\quad+ \,I_0^{Interface}\left[ {1 - e^{\frac{{ - \left( {d_{is} + d_{if}} \right)}}{\lambda }}} \right]e^{\frac{{ - \left( {d - d_{if}} \right)}}{\lambda }}\end{array}$$

Thus, the intensity of the interface state spectrum can be determined as

7 $$I_0^{Interface} = \frac{{I^{expt}(d) - \left\{ {I_0^{Substrate}e^{\frac{{ - \left( {d + d_{is}} \right)}}{\lambda }} + I_D^{Film}\frac{{\left[ {1 - e^{\frac{{ - \left( {d - d_{if}} \right)}}{\lambda }}} \right]}}{{1 - e^{\frac{{ - D}}{\lambda }}}}} \right\}}}{{\left[ {1 - e^{\frac{{ - \left( {d_{is} + d_{if}} \right)}}{\lambda }}} \right]e^{\frac{{ - \left( {d - d_{if}} \right)}}{\lambda }}}}$$

#### Step 3: (Calculating interface states using different film thickness)

Once the parameters λ, and film thickness $(d,D)$ are determined, the only variable parameters in Eq. (7) are *d*_*is*_ and *d*_*if*_, which are the components of the interface layer thickness contributed by the substrate and the film, respectively. For certain interface model structure *d*_*is*_ and *d*_*if*_ (Fig. 4), one can calculate a set of $I_0^{Interface}$ data ($I_{0[d_1]}^{Interface}$, $I_{0[d_2]}^{Interface}$, $I_{0[d_3]}^{Interface}$, …) using the available experimental data $I^{expt}(d)$ at different *d* $(d_1,d_2,d_3 \ldots )$, based on Eq. (7). Different valuables of *d*_*is*_ and *d*_*if*_ for interface layer thickness can be used to obtain different $I_0^{Interface}$ sets of data. The most likely interface layer structure *d*_*is*_ and *d*_*if*_ would correspond to the particular $I_0^{Interface}$ set of data, in which case, $I_{0[d_1]}^{Interface}$, $I_{0[d_2]}^{Interface}$, $I_{0[d_3]}^{Interface}$… are similar to each other.

#### Step 4: (Determining interface state spectrum based on the best-fit interface layer model)

Once the best-fit interface layer model is determined, the interface state spectrum can be finally calculated by averaging the $I_0^{Interface}$ set of data with the corresponding values of the *d*_*is*_ and *d*_*if*_ parameters for the best-fit model.

### Case studies of the quantitative modeling of spectra for interface states

**Fig. 5:**
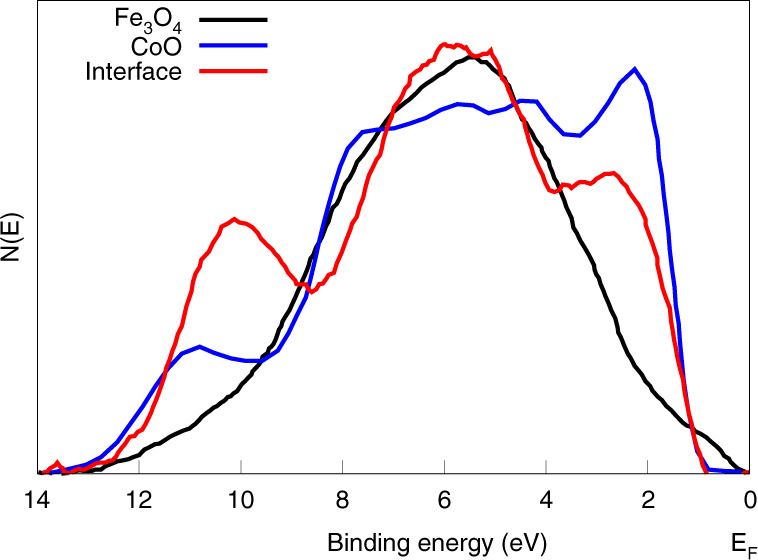
The determined spectrum of interface states between Fe_3_O_4_-CoO based on the quantitative modeling method, compared with the substrate spectrum of Fe_3_O_4_ and the film spectrum of CoO. Reproduced with permission (adapted from^82^). Copyright 2018, American Physical Society

The above-mentioned quantitative modeling has been used to study the interfaces between Fe_3_O_4_ and other transition-metal oxides, specifically NiO and CoO. All of these oxides are of significant interest in spintronics. In particular, Fe_3_O_4_–NiO and Fe_3_O_4_–CoO have been proposed as an ingredient in all-oxide tunneling spin valves^98^. Fe_3_O_4_ is a metallic ferrimagnet, and both NiO and CoO are insulating antiferromagnets. The exchange biasing effect^99–103^ in which the hysteresis loop of a ferro- or ferrimagnet is shifted asymmetrically along the field axis when in contact with an antiferromagnetic material, has been observed for both interfaces, making them interesting for spintronics. NiO and CoO have the same rocksalt crystal structure, and, although Fe_3_O_4_ has the inverse spinel structure, both structures share a common face-centered-cubic oxygen sublattice, where the lattice mismatch is only 0.55% between Fe_3_O_4_ and NiO and 1.45% between Fe_3_O_4_ and CoO. Despite the fact that NiO and CoO have very similar bulk electronic properties, it is interesting that, the Fe_3_O_4_ (001) − NiO (001) interface exhibits a sharp interface without obvious interface electronic state^88^, while the Fe_3_O_4_ (001)–CoO (001) interface displays non-trivial electronic state and the interface state spectrum was determined using the above mentioned quantitative modeling by comparing two interface layer models^82^. In one case, the interface layer consisted of one monolayer of the substrate Fe_3_O_4_ plus one monolayer of the film CoO; in the other case, the interface layer consisted of only one monolayer of the film CoO. The determination of the better-fit interface model was based on the observation of the degree of similarity among the generated three spectra of $I_{0[d_1]}^{Interface}$, $I_{0[d_2]}^{Interface}$, $I_{0[d_3]}^{Interface}$, using each model. It was concluded that the first case where the interface layer consists of one monolayer of the substrate Fe_3_O_4_ plus one monolayer of the film CoO is closer to the actual case. The interface states spectrum determined using the best-fit model is shown in Fig. 5.

## Discussions

Although the quantitative modeling of experimental PES spectra as presented above is a simplified version to some extent, it captures the main feature of the interface states without losing the generality. More technical details or physics phenomenon can be included in the framework of aforementioned analysis procedure by modifying corresponding terms. One prospect related to PES technique is the photoelectron diffraction (PED) effects^104–106^. Due to the PED effects, the PES intensity can be modulated depending on the electron emission angles (polar and azimuthal) or energy of incident x-ray source^104,105^. For the angle-dependent PES, the formula can be further modified to include the effects of x-ray incident angles and photoelectron collection angles on the emission intensity.

The other prospect is related to the structural and compositional nature of the interface region. For example, at oxide/oxide interfaces, cation mixing often occurs. In fact, our quantitative modeling procedure as presented above is valid for general electronic interface states, which is caused either by electronic reconstruction or by atomic re-arrangement including atom mixing at interface. Of course, in order to fully investigate the origin of the interface states, such as the composition and structure of the interface, one may combine quantitative spectra modeling with other characterization techniques such as synchrotron radiation XAS or scanning transmission electron microscopy (e.g., quantitative electron diffraction^107–109^ or EELS, etc) or theoretical computations including first-principles calculations based on density functional theory to perform an integrated study^109,110^.

## Conclusion and future aspect

In conclusion, this review article summaries several approaches that have been adopted to extend the application of surface-sensitive photoemission techniques to buried interfaces. A quantitative method is reviewed to extract the electronic states at the embedded interface between two functional materials, based on the thickness-dependent experimental photoemission spectra, which is one of the important techniques to determine the valance band the electronic structure at the interface. The quantitative model method also serves as an efficient and effective approach to determine the interface layer model involving the component layers from the substrate and the film, respectively.

It is expected that this quantitative modeling method could be extended to other electronic states probes and would have a broad application in probing interfacial electronic states, which are crucial for device performance. The current modeling method is based on the assumption that the film is deposited on the substrate surface following the ideal layer-by-layer growth mode. In the future, this modeling method can be further developed to take into account other possible growth modes.

## Appendix 1. List of surface analysis acronyms^20^

**Table:**

1. Electron Excitation
AES, Auger electron spectroscopy
BIS, Bremsstrahlung isochromat spectroscopy (or ILS, ionization loss spectroscopy)EDXS, Energy-dispersive X-ray spectroscopy
EELS, Electron energy loss spectroscopy
EFTEM, Energy-filtered transmission electron microscopy
ESD, Electron-stimulated desorption (or EID, electron-induced desorption)
ESDIAD, Electron-stimulated desorption ion angular distribution
IPES, Inverse photoemission spectroscopy
LEED, Low-energy electron diffraction
RHEED, Reflection high-energy electron diffraction
SXAPS, Soft X-ray appearance potential spectroscopy (or APS, appearance potential spectroscopy)
SAM, Scanning Auger microscopy
SEM, Scanning Electron microscopyEF-TEM, Energy-filtering transmission electron microscopy
2. Ion Excitation
ERDA, Elastic recoil detection analysis
GDMS, Glow discharge (GD) mass spectrometry
GD-OES, Glow discharge (GD) optical emission spectroscopy (OES)
IAES, Ion (excited) Auger electron spectroscopy
IBSCA, Ion beam spectrochemical analysis (or SCANIIR, surface composition by analysis of neutral and ion impact radiation or BLE, bombardment induced light emission)
INS, Ion neutralization spectroscopy
LEIS, Low-energy ion scattering (or ISS, Ion-scattering spectroscopy)
NRA, Nuclear reaction analysis
RBS, Rutherford back-scattering spectroscopy (or HEIS, high-energy ion scattering)
SIMS, Secondary-ion mass spectrometry
(SSIMS, static secondary-ion mass spectrometry)
(DSIMS, dynamic secondary-ion mass spectrometry)
SNMS, Secondary neutral mass spectrometry
3. Photon Excitation
ELL, Ellipsometry
LA, Laser ablation
LIBS, Laser-induced breakdown spectroscopy (or LIPS, Laser-induced plasma spectroscopy)
RAIRS, Reflection-absorption infrared spectroscopy (or IRRAS, infrared reflection-absorption spectroscopy, or IRAS, infrared absorption spectroscopy, or ERIRS, external reflection infrared spectroscopy)
ATR, Attenuated total reflection
FTIR, Fourier transform infred spectroscopy
SERS, Surface-enhanced Raman scattering
SFG, Sum frequency generation
SHG, (optical) Second-harmonic generation
SNOM, Scanning near-field optical microscopyTXRF, Total reflection X-ray fluorescence (XRF) analysis
UPS, Ultraviolet photoelectron spectroscopy
XPS, X-ray photoelectron spectroscopy (or ESCA, electron spectroscopy for chemical analysis)
XRD, X-ray diffraction
XAFS, X-ray absorption fine structure
4. Neutral Excitation
FABMS, Fast-atom bombardment mass spectrometry
5. Thermal Excitation
TDS, Thermal desorption spectroscopy
6. High-Field Excitation
AP, Atom probe
FIM, Field ion microscopy
IETS, Inelastic electron tunneling spectroscopy
STM, Scanning tunneling microscopy
STS, Scanning tunneling spectroscopy
7. Mechanical Force
AFM, Atomic force microscopy
